# Tributyrin Supplementation Protects Immune Responses and Vasculature and Reduces Oxidative Stress in the Proximal Colon of Mice Exposed to Chronic-Binge Ethanol Feeding

**DOI:** 10.1155/2018/9671919

**Published:** 2018-08-19

**Authors:** B. Glueck, Y. Han, G. A. M. Cresci

**Affiliations:** ^1^Lerner Research Institute, Inflammation and Immunity, Cleveland Clinic, Cleveland, OH, USA; ^2^Pediatric Institute, Gastroenterology, Cleveland Clinic, Cleveland, OH, USA; ^3^Digestive Disease & Surgery Institute, Gastroenterology, Hepatology & Nutrition Cleveland Clinic, Cleveland, OH, USA

## Abstract

Excessive ethanol consumption causes adverse effects and contributes to organ dysfunction. Ethanol metabolism triggers oxidative stress, altered immune function, and gut dysbiosis. The gut microbiome is known to contribute to the maintenance of intestinal homeostasis, and disturbances are associated with pathology. A consequence of gut dysbiosis is also alterations in its metabolic and fermentation byproducts. The gut microbiota ferments undigested dietary polysaccharides to yield short-chain fatty acids, predominantly acetate, propionate, and butyrate. Butyrate has many biological mechanisms of action including anti-inflammatory and immunoprotective effects, and its depletion is associated with intestinal injury. We previously showed that butyrate protects gut-liver injury during ethanol exposure. While the intestine is the largest immune organ in the body, little is known regarding the effects of ethanol on intestinal immune function. This work is aimed at investigating the effects of butyrate supplementation, in the form of the structured triglyceride tributyrin, on intestinal innate immune responses and oxidative stress following chronic-binge ethanol exposure in mice. Our work suggests that tributyrin supplementation preserved immune responses and reduced oxidative stress in the proximal colon during chronic-binge ethanol exposure. Our results also indicate a possible involvement of tributyrin in maintaining the integrity of intestinal villi vasculature disrupted by chronic-binge ethanol exposure.

## 1. Introduction

Excessive ethanol consumption causes damage to various organs and systems. While the liver is the primary target of ethanol's detrimental effects, the brain, pancreas, lungs, intestine, and the immune system are also known to be affected. It is generally recognized that lipid peroxidation, immune damage, and antioxidant defenses may play an important role in the pathogenesis of ethanol-induced cellular injury [1]. Ethanol promotes the generation of superoxide anion and hydrogen peroxide, and these byproducts contribute to endothelial dysfunction, vasoconstriction, and hypertension [2].

Both acute ethanol and chronic ethanol interfere with multiple aspects of innate immune responses resulting in chronic alcoholics having an increased risk and severity of infections. This association has been demonstrated with several types of infections including pulmonary [3], hepatitis C [4], and human immunodeficiency virus [5]. Ethanol suppresses tissue recruitment of polymorphonuclear neutrophils (PMNs) during infection and inflammation, which can impact susceptibility to infection, decrease bacterial clearance, and increase mortality from pneumonia [6]. Ethanol abuse alters granulopoiesis [3] and inhibits cell division and differentiation of precursor cells into granulocytes [3]. Ethanol also compromises phagocytic activity of blood monocytes and resident macrophages [7] and their ability to adhere to cells and to engage in intracellular microbe killing [8]. Ethanol impairs natural killer cell activity, decreasing their ability to destroy their target cells [9, 10].

Recently recognized is the link between ethanol consumption and intestinal bacterial overgrowth and dysbiosis in both animal and human studies [11–15]. The intestinal tract, the largest immune organ in the body, is comprised of more immunoglobulin-producing cells compared to bone marrow, spleen, and lymph nodes and contains resident and infiltrating immune cells [16]. Intestinal macrophages are located in the lamina propria within the mucosa and therefore in close proximity to the epithelial layer [17]. The presence of immune cells varies throughout the gastrointestinal tract, with higher predominance of macrophages localized in the colon compared to the small intestine in rodents and humans [18, 19]. This dynamic immune organ is on constant surveillance to maintain intestinal homeostasis by regulating immune responses not only to ingested pathogens but also to the trillions of commensal microorganisms comprising the gut microbiome [20]. While the gut microbiota is involved with digestion and metabolism, its important regulatory role in inflammation and immunity is also greatly appreciated [20]. Therefore, gut microbial disruption influences intestinal homeostasis.

As a consequence to gut dysbiosis, alterations in gut microbial metabolic and fermentation byproducts occur, such as depletions of the short-chain fatty acids, acetate, propionate, and butyrate. Butyrate plays many well-documented roles in the intestine including serving as the primary fuel source for the colonocyte, regulating water and electrolyte absorption and gene expression, providing support of the epithelial barrier, modulating visceral sensitivity and intestinal motility, and ameliorating mucosal inflammation and oxidative stress [21].

Tributyrin is a structured lipid with 3 butyrate molecules esterified to glycerol. Upon oral ingestion, tributyrin is hydrolyzed by pancreatic and gastric lipases, yielding glycerol and 3 butyrate molecules. Tributyrin is safe when provided at lower doses, but can be cytotoxic at higher doses (e.g., *in vivo*, ≥10.3 g/kg; *in vitro*, >10 mM) [22–26]. Our previous work in animal models of antibiotic treatment [26] and ethanol exposure [27, 28] demonstrates several beneficial effects of tributyrin supplementation during these treatments. In these studies, providing tributyrin orally protects against intestinal barrier losses with preservation of the tight junction protein complex and preserves expression of several genes and proteins involved with water and electrolyte balance, butyrate transport, and inflammation [26–28]. When provided during animal models of acute and chronic-binge ethanol exposure, tributyrin supplementation not only preserves the intestinal barrier but also is hepatoprotective [27, 28].

Following a physiologic insult, an immune response needs to be adequately mounted and resolved for proper return of organ homeostasis and function. Investigations in the effects of ethanol exposure on intestinal immune function are limited. Due to the positive effects of tributyrin we found during chronic-binge ethanol exposure on gut-liver injury, and the beneficial effects of butyrate on inflammation and immunity, we aimed to determine whether tributyrin influenced intestinal immune responses in a mouse model of chronic-binge ethanol exposure. Here, we present the effects of tributyrin supplementation on innate immune responses, vasculature, and oxidative stress in the proximal colon following chronic-binge ethanol exposure in mice.

## 2. Materials and Methods

### 2.1. Materials

Glyceryl tributyrate (tributyrin) and sodium butyrate came from Sigma-Aldrich (St. Louis, MO, USA). Interleukin 1 beta came from Sino Biological (IL-1*β*; Beijing, China). Pair-fed control diet and Lieber-DeCarli high-fat ethanol diet came from Dyets Inc. (Bethlehem, PA, USA). All primers for quantitative real-time reverse transcription polymerase chain reaction (qRT-PCR) were synthesized by Integrated DNA Technologies (Coralville, IA, USA). The following are primary antibodies: antigranulocye colony-stimulating factor (G-CSF), anti-Von Willebrand factor (vWF), and leukocyte common antigen, CD45 (Clone I3/2.3), came from Abcam (Cambridge, MA); anti-neutrophil (NIMP14) antibody came from Novus Biologicals (Littleton, CO); C3b/iC3b/C3c (C3b) came from Hycult Biotechnology (Uden, Netherlands); anticluster of differentiation, CD68, was from AbD Serotec (Raleigh, NC); and antiplatelet endothelial cell adhesion molecule (PECA1)/CD31 was from Genetex (Irvine, CA).

### 2.2. Methods

Eight- to 10-week-old female C57BL/6J mice were purchased from Jackson Laboratories (Bar Harbor, ME, USA). Mice were housed in standard microisolator cages (two animals per cage) and fed standard laboratory chow (rodent diet #2918, Harlan-Teklad, Madison, WI, USA) during a 1-week acclimation period prior to being fed a liquid diet. Ten-week-old chow-fed female C3^−/−^ mice on a C57BL/6J background were a gift from Feng Lin, PhD (Cleveland Clinic). The Institutional Animal Care and Use Committee approved all animal procedures.

#### 2.2.1. Chronic-Binge Ethanol Feeding and Tributyrin Provision

Weight-matched animals were randomly assigned so that each treatment group was within 0.5 gm of each other and then adapted to a control liquid diet for 5 days. Following adaption, mice were allowed ad libitum access to either a 5% *v*/*v* (27% total kcal) ethanol-containing diet or a pair-fed diet that isocalorically substituted maltose dextrin for ethanol for 10 days. Diets were made fresh every other day and supplemented with tributyrin (5 mM) or glycerol (5 mM) over the 10 days of ethanol feeding. On day 11, mice were gavaged with a 5 g/kg dose of ethanol or isocaloric maltose. Tributyrin (7.5 mg) or glycerol (2.3 mg) was included in the gavage at a concentration of 2.5 mM. Mice were anesthetized and euthanized 9 h post-gavage. The intestine was dissected and frozen in optimal cutting temperature (Sakura Finetek USA, Torrance, CA, USA), snap frozen in liquid nitrogen, or stored in RNAlater (Ambion, Austin, TX, USA) for further analysis.

#### 2.2.2. Immunohistochemistry

Proximal colon sections frozen in optimal cutting temperature (OCT) compound were used for immunostaining of proteins expressed by immune cells (CD68; leukocyte common antigen, CD45), endothelial cells (platelet endothelial cell adhesion molecule-1 (PECAM-1) or CD31; von Willebrand factor (vWF)), and granulocyte colony-stimulating factor (G-CSF) as previously described [29]. No specific immunostaining was seen in sections incubated with phosphate-buffered saline (PBS) versus the primary antibody. Slides were coded before examination, and a single investigator blinded to treatments viewed them. All images presented represent at least three images per tissue section and four to six mice per experimental condition. Semiquantification of positive staining was performed using Image Pro Plus software (Media Cybernetics, Silver Spring, MD, USA). Images were taken under fluorescence microscope (Leica DM2000).

#### 2.2.3. Quantitative Real-Time Reverse Transcription PCR

Total RNA was isolated from the proximal colon from at least four to six mice per experimental condition, and 2 *μ*g of total RNA was reverse-transcribed as previously described [29]. Real-time PCR amplification was performed using Power SYBR qRT-PCR kits (Applied Biosystems, Foster City, CA) on a QuantStudio 5 analyzer (Applied Biosystems) for the following primers: heme oxygenase 1 (HO-1), nicotinamide adenine dinucleotide phosphate (NADPH) oxidase 1 (NOX1), superoxide dismutase 2 (SOD2), thioredoxin 1 (TRX1), platelet-derived growth factor subunit b (PDGFb), transforming growth factor beta 1 (TGF*β*1), matrix metallopeptidase 9 (MMP9), arginase 1 (ARG1), lymphocyte antigen 6 complex (Ly6c), CD68, neutrophil elastase (ELANE), and inducible nitric oxide synthase (iNOS). Glyceraldehyde 3-phosphate dehydrogenase (GAPDH) or 18S was used as a housekeeping gene (see Table 1 for sequences). A relative amount of target messenger RNA (mRNA) was determined using the comparative threshold (Ct) method by normalizing target mRNA Ct values to those of 18S or GAPDH and represented as fold change relative to pair-fed-treated mice.

#### 2.2.4. Caco-2 Cell Culture and Treatments

The human epithelial colorectal adenocarcinoma cell line (Caco-2; ATTC, Manassas, VA, USA) was cultured in Dulbecco's modified Eagle's medium (Gibco BRL Products, Grand Island, NY, USA) supplemented with 4.5 g/L glucose, 10% fetal bovine serum (Invitrogen/Gibco), 1% antibiotic solution (penicillin G, streptomycin B; Gibco BRL Products, Grand Island, NY, USA), and 1% nonessential amino acids. Cells were grown in 75 cm^2^ T-flasks (Fisher, St. Louis, MO, USA) at 37°C and 5% CO_2_. The medium was changed thrice a week, and cells were passaged every 5 to 7 days at 80% confluency. Cells were used at passages 30 and 31 for this experiment.

Monolayers were harvested at confluency by washing the cells with PBS followed by trypsin-EDTA solution. Caco-2 cells were seeded at 0.2 × 10^6^/well into a 24-well plate. The medium was changed every 3 days, and the cell monolayer allowed differentiating for 7 days. Cells were pretreated with serum-free medium with or without sodium butyrate (5 mM) for 18 h followed by a challenge with 25 mM ethanol and/or 10 ng/mL IL-1*β* for 3 h. Butyrate remained in the appropriate pretreated wells for the duration of the challenge. Treatments were performed in duplicate and repeated four times.

#### 2.2.5. IL-8 ELISA Assay

Caco-2 cells were pretreated with sodium butyrate and challenged with ethanol and/or IL-1*β* as described above. The extracellular media was collected at the end of the experiment and analyzed for the presence of the IL-8 cytokine by enzyme-linked immunosorbent assay (ELISA; Eagle Biosciences, Nashua, NH, USA) as per the manufacturer's instructions.

#### 2.2.6. Statistical Analysis

All data are expressed the mean ± standard error of the mean (SEM) and *n* = 4–6 mice per treatment groups and *n* = 4 experiments for cell culture. The Student *t*-test was used for parametric analysis of 2 groups; analysis of variance was used for comparison of multiple groups, with Tukey's post hoc test for multiple comparisons. Data were log-transformed to obtain a normal distribution as needed. Statistical significance was defined as *p* < 0.05. The analysis was performed using the Prism software Version 5.02 (GraphPad Software, San Diego, CA, USA).

## 3. Results

### 3.1. Tributyrin Maintained Ethanol-Induced Dampening of Immune Cell Density in Proximal Colon

**Figure 1:**
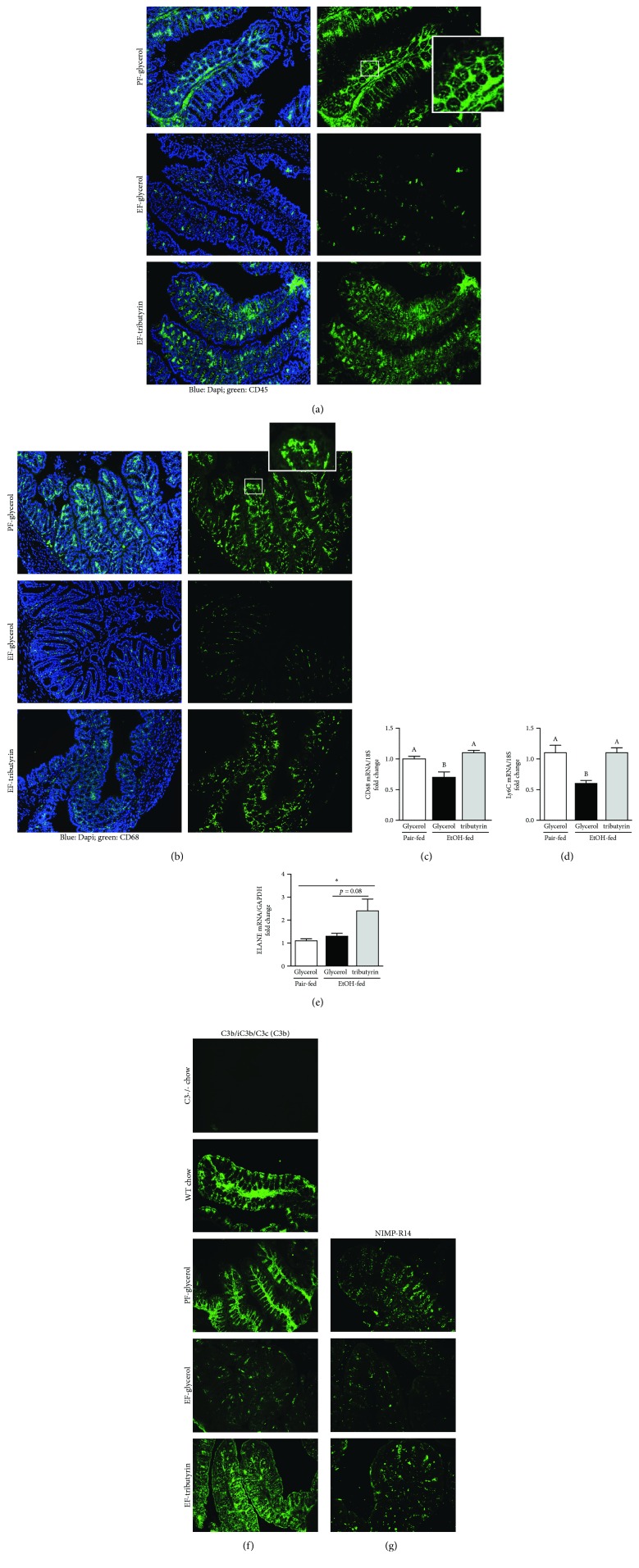
Effects of tributyrin on presence of immune cells in proximal colon following chronic-binge ethanol exposure. Mice were fed an ethanol-containing (5% *v*/*v*) liquid diet or pair-fed a diet with maltose-dextrin isocalorically substituted for ethanol for 10 days. Diets were supplemented with glycerol or tributyrin (5 mM). Mice were then treated with a single 5 g/kg gavage of ethanol the next day containing glycerol or tributyrin (2.5 mM). At 9 h post-gavage, the proximal colon was collected and used to prepare RNA or embedded in optimal cutting temperature compound (OCT) for histology. (a) CD45 (green), (b) CD68 (green), (f) C3b/iC3b/C3c, and (g) NIMP-R14 were visualized by immunohistochemistry in sections of proximal colon frozen in OCT. Images were acquired using a 10x or 20x objective. A selected area was cropped and enlarged. (c–e) Expression of CD68, Ly6c, and ELANE mRNA was detected in the proximal colon using quantitative real-time reverse transcription polymerase chain reaction. (f) In addition to pair-fed and ethanol-treated mice, proximal colon frozen in OCT from age- and gender-matched C57BL/6 and C3^−/−^ mice on a C57BL/6 background were stained for expression of C3b/iC3b/C3c. C3b-positive staining was visualized in wild-type mice similarly to that of pair-fed mice, and, as expected, C3^−/−^ were negative for positive C3b staining. Images are representative of at least replicate images captured per mouse in four to six mice per treatment group. Data are mean ± standard error of the mean (SEM). Values with different alphabetical superscripts were significantly different from each other; *p* < 0.05 and ^∗^*p* < 0.05.

Mice exposed to ethanol for 10 days followed by a single ethanol gavage and euthanized 9 hours later exhibited less positive staining of markers of innate immunity (CD45, CD68, C3b, and NIMP-R14) (Figures 1(a), 1(b), 1(f), and 1(g)) and decreased mRNA expression of macrophages (CD68, Ly6c) (Figures 1(c) and 1(d)) in the proximal colon. Mice supplemented with tributyrin exhibited similar immunoreactive staining intensity of immune cells, CD45, CD68, and mRNA levels of CD68 and Ly6c as visualized in pair-fed mice (Figures 1(a)–1(d)). Neutrophil expression, measured by NIMP-R14, was depleted in ethanol-only-treated animals compared to pair-fed and tributyrin-supplemented mice (Figure 1(g)), and ELANE mRNA was highest in the tributyrin-treated group (Figure 1(e)). With C3b staining intensity, the expression was similar in tributyrin-treated animals to that of pair-fed and wild-type chow-fed mice, but negative in C3^−/−^ mice and diminished in ethanol-only-treated mice (Figure 1(f)). The positive mucosal staining of markers of innate immunity is notably confined to the lamina propria where blood and lymph vessels, eosinophils, lymphocytes, and plasma cells are localized and, except for C3b, excluded from the epithelium.

### 3.2. Tributyrin Supported Intestinal Neutrophil Presence in the Proximal Colon

**Figure 2:**
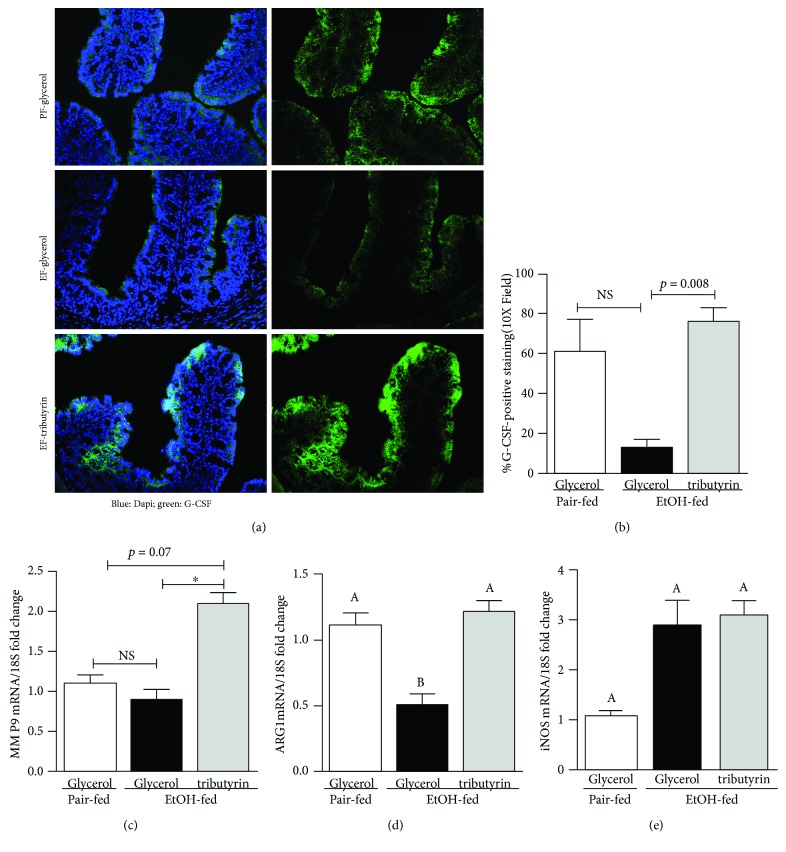
Effect of tributyrin on neutrophil and macrophage regulators in the proximal colon following chronic-binge ethanol exposure. Mice were treated as described in Figure 1, and the proximal colon was excised and used to prepare RNA or embedded in OCT for histology. (a) G-CSF (green) was visualized by immunohistochemistry in sections of proximal colon frozen in OCT. Images shown were acquired using 20x objective and are representative of at least replicate images captured per mouse in four to six mice per treatment group. (b) Images acquired using a 10x objective were quantified for G-CSF-positive areas using Image Pro Plus software and analyzed. (c–e) Expression of MMP9, Arg1, and iNOS mRNA was detected in the proximal colon using quantitative real-time reverse transcription polymerase chain reaction. Data are mean ± SEM. Values with different alphabetical superscripts were significantly different from each other; *p* < 0.05 and ^∗^*p* < 0.05; NS = not significant.

Neutrophils, the first line of innate immune system defense, are produced in the bone marrow by granulopoiesis, and granulocyte colony-stimulating factor (G-CSF) is the principle cytokine which regulates this process [30]. Since we noted a variance in immune cells between treatment groups, we investigated the presence of G-CSF in the proximal colon. While G-CSF staining was noted throughout the proximal colon, it was less visible in mice exposed to ethanol (Figure 2(a)). Positive G-CSF staining was similar between animals supplemented with tributyrin and those pair-fed (Figure 2(b)).

Neutrophil granules contain large amounts of matrix metalloproteinase 9 (MMP9). Matrix metalloproteinases are the group of proteolytic enzymes which degrade the extracellular matrix. By breaking down surrounding tissue, MMPs facilitate infiltration of circulating cells, such as monocytes, into tissue. Therefore, we assessed MMP9 mRNA expression in the proximal colon. Mice supplemented with tributyrin and exposed to ethanol had increased mRNA expression of MMP9 in the proximal colon compared to mice not supplemented with tributyrin, both pair-fed and ethanol-exposed (Figure 2(c)).

Alternatively activated macrophages (M2) are involved with phagocytosis, production of the extracellular matrix, and chemotactic and angiogenic factors [31]. It is common to classify macrophages into one of two subtypes, M1 or M2, based on surface receptor expression they produce. In the mouse, arginase 1 (Arg1) is considered a prototypic marker of M2 macrophages and tissue resident macrophages constitutively express Arg1, and iNOS is a marker for M1 macrophages [32, 33]. We tested for the mRNA expression of iNOS and Arg1 in the proximal colon as a means to identify presence of M1 and M2 macrophages, respectively. Exposure to ethanol reduced Arg1 mRNA expression in the proximal colon (Figure 2(d)), but tributyrin supplementation normalized that effect. No difference was found in iNOS mRNA expression between treatment groups (Figure 2(e)).

### 3.3. Butyrate Normalized IL-8 Expression in Caco-2 Cell Monolayers

**Figure 3:**
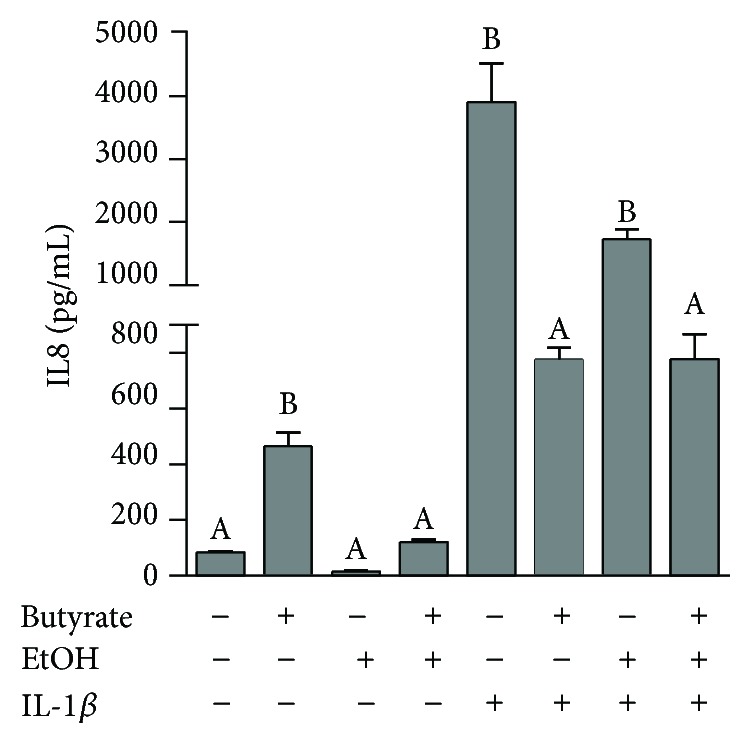
Effect of butyrate on secreted IL-8 from Caco-2 monolayers stimulated with IL-1*β* and ethanol. Human intestinal epithelial cells (Caco-2) were grown to confluency in 24-well plates, and monolayers were allowed to differentiate for 7 days. Cells were pretreated ± sodium butyrate (5 mM) for 18 h and then challenged with 25 mM ethanol and/or 10 ng/mL IL-1*β* for 3 h. Extracellular media was then collected and analyzed for IL-8 by ELISA. Treatments were performed in duplicate and repeated four times. Data are mean ± SEM. Values with different alphabetical superscripts were significantly different from each other, *p* < 0.05.

IL-1*β*, known to initiate and amplify inflammation, is a cytokine released by various cell types including monocytes, macrophages, neutrophils, and endothelial cells. *In vitro*, IL-1*β* has been shown to increase chemokine IL-8 from intestinal epithelial cells [34]. Also known as neutrophil chemotactic factor, IL-8 is released from intestinal epithelial cells and induces both chemotaxis in target cells as well as phagocytosis and is also a promoter of angiogenesis [35, 36]. In order to test butyrate's direct effect on IL-8 release from intestinal epithelial cells, we pretreated Caco-2 cells with butyrate and then exposed them to IL-1*β* and/or ethanol. As expected, in response to IL-1*β*, there was a large induction of IL-8 release from Caco-2 cells (Figure 3). Compared to untreated Caco-2 cells, butyrate treatment induced and ethanol mildly reduced IL-8 release, although this reduction did not approach significance (Figure 3). Compared to the induction of IL-8 by IL-1*β* treatment, cotreatment with ethanol mildly reduced IL-8 secretion, although this reduction did not approach significance (Figure 3). However, when Caco-2 cells were treated with butyrate and stimulated with IL-1*β* and/or ethanol, IL-8 levels in supernatants were significantly reduced with butyrate compared to IL-1*β* and/or ethanol-stimulated Caco-2 cells without butyrate treatment (Figure 3).

### 3.4. Tributyrin Modulated Ethanol-Induced Oxidative Stress Responses in the Proximal Colon

**Figure 4:**
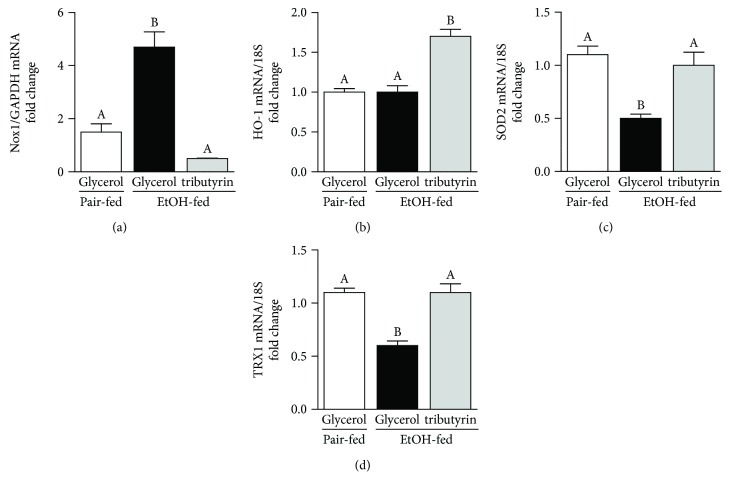
Effects of tributyrin on expression of pro- and antioxidant mediators in the proximal colon following chronic-binge ethanol exposure. Mice were treated as described in Figure 1, and the proximal colon was excised and used to prepare RNA. (a–d) Expression of NOX1, HO-1, SOD2, and TRX1 mRNA was detected in the mouse proximal colon using quantitative real-time reverse transcription polymerase chain reaction. Values represent means ± SEM. *n* = 4–6 mice per treatment group. Values with different alphabetical superscripts were significantly different from each other, *p* < 0.05.

Butyrate is known to have antioxidant properties by modulating production of antioxidant enzymes [37]. NADPH/NOX1 is one of the major enzymes involved with ethanol metabolism and generation of reactive oxygen species (ROS). Thioredoxin (TRX1) and superoxide dismutase 2 (SOD2) serve key antioxidant roles. Upon evaluation of the effect chronic-binge ethanol exposure had on the generation and resolution of ROS, compared to mice treated with tributyrin and those pair-fed, we found that NOX1 mRNA expression was induced in mice only exposed to ethanol, and SOD2 and TRX1 mRNA was reduced (Figures 4(a), 4(c), and 4(d)). Heme oxygenase 1 induction provides potent cytoprotective effects on various models of oxidative damages and stresses [38]. Tributyrin treatment induced HO-1 mRNA levels in the presence of ethanol, whereas levels were similar in groups not treated with tributyrin (Figure 4(b)).

### 3.5. Tributyrin Preserved Vasculature within the Proximal Colon during Ethanol Exposure

**Figure 5:**
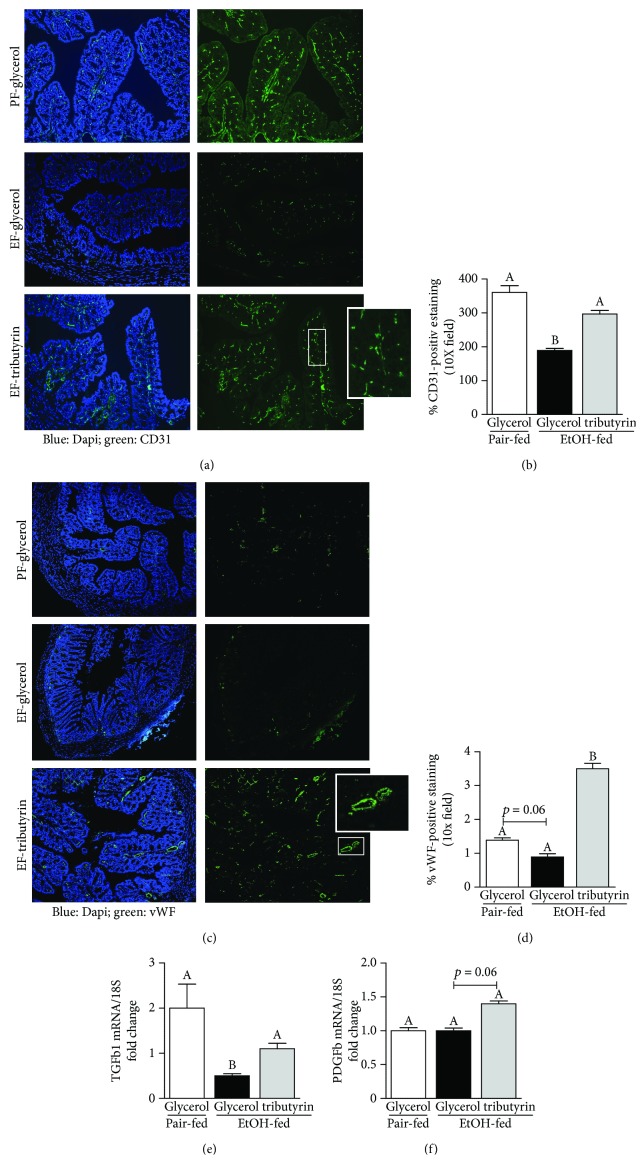
Effect of tributyrin on vasculature in the proximal colon following chronic-binge ethanol exposure. Mice were treated as described in Figure 1, and the proximal colon was excised and used to prepare RNA or embedded in OCT for histology. (a) CD31 (green) and (c) vWF (green) were visualized by immunohistochemistry in sections of proximal colon frozen in OCT. All images were acquired using a 10x objective. A selected area was cropped and enlarged. Images are representative of at least replicate images captured per mouse in four to six mice per treatment group. (b, d) CD31- and vWF-positive areas were quantified using Image Pro Plus software and analyzed. (e, f) Expression of TGF*β*1 and PDGFb mRNA was detected in the proximal colon using quantitative real-time reverse transcription polymerase chain reaction. Data are mean ± SEM. Values with different alphabetical superscripts were significantly different from each other, *p* < 0.05.

Metabolism of ethanol can cause hypoxia. Reactive oxygen species derived from NADPH oxidase are involved with the development of tissue dysfunction induced by ethanol [39, 40]. Activity of NADPH oxidase is involved with ethanol-induced hypertension and ROS generation in the vasculature [2]. Ideally in order to facilitate recovery, angiogenesis follows tissue ischemia and hypoxia. The presence of monocytes and neutrophils enables paracrine signaling between the endothelium and perivascular cells to create space for growing vessels [41]. Ethanol is known to inhibit neutrophil migration [13, 42–44]. The noted variation between ethanol treatment groups in the presence and distribution pattern of monocytes in the proximal colon (Figures 1(a) and 1(b)), resembling that of the vasculature of the villi, led us to investigate for markers of endothelial cells (CD31 and vWF) by immunohistochemistry for potential ethanol-induced derangements. Both CD31- and vWF-positive staining was depleted in mice only receiving ethanol (Figures 5(a)–5(d)). Staining intensity for CD31 was robust and similar between pair-fed mice and those supplemented with tributyrin (Figures 5(a) and 5(b)). In mice supplemented with tributyrin, staining intensity for vWF was higher compared to those pair-fed or only exposed to ethanol (Figures 5(c) and 5(d)). Evaluation of mRNA expression of TGF*β*1 and PDGFb, growth factors involved with angiogenesis, found lower expression of TGF*β*1 in animals only treated with ethanol (Figure 5(e)) and trended towards increased PDGFb in mice supplemented with tributyrin (Figure 5(f)).

## 4. Discussion

Ethanol exposure is known to disrupt the gut microbiota which consequentially affects the production of beneficial fermentation byproducts, including short-chain fatty acid butyrate [13, 45, 46]. Gut dysbiosis is associated with altered immune responses and disruption in intestinal homeostasis. In the present study, we find that chronic-binge ethanol exposure negatively impacts intestinal innate immune responses, markers of oxidative stress and vasculature in the proximal colon. Here, for the first time we provide evidence of a remarkable beneficial effect of prophylactic tributyrin supplementation in supporting not only the presence of immune cells but also antioxidant defenses and intestinal vasculature in mice exposed to chronic-binge ethanol treatment.

Ethanol metabolism induces oxidative stress. As ethanol cannot be excreted, it is metabolized primarily in the liver, as well as in extrahepatic tissues including the intestine [47]. The major enzyme pathways which metabolize ethanol into acetaldehyde and then acetate are alcohol dehydrogenase/aldehyde dehydrogenase and the microsomal ethanol-oxidizing system catalyzed by cytochrome P450 2E1 (CYP2E1) [47]. The ethanol-induced CYP2E1 pathway metabolizes ethanol while oxidizing biosynthetic reducing power, NADPH to NADP^+^. Because this pathway uses oxygen, free radicals that damage tissues are generated. Additionally, since NADPH is consumed, the potent endogenous antioxidant glutathione cannot be regenerated, further exacerbating oxidative stress [47].

Butyrate, known to have anti-inflammatory and antioxidant properties, reduces levels of reactive oxygen species in vascular smooth muscle cells by modulating the redox state by inducing glutathione-S-transferase [48]. Aguilar et al. demonstrated a reduction in superoxide production and protein nitrosylation with butyrate supplementation in a mouse model of reduced atherosclerotic development [49]. In this model, stimulated peritoneal macrophages had a lower free radical release when pretreated with butyrate, which was related to a reduction in NADPH oxidase and inducible nitric oxide synthase. Marchi et al. recently showed that ethanol-induced hypertension is mediated by NADPH oxidase and that NOX1 expression is related to the generation of reactive oxygen species by ethanol [2]. In our chronic-binge ethanol exposure model, mice only receiving ethanol had induced mRNA levels of NOX1 and reduced mRNA levels of antioxidant genes. More importantly, tributyrin supplementation was able to mitigate the prooxidant effects of chronic-binge ethanol exposure in the proximal colon, a region naturally physiologically hypoxic.

As the innate immune response is the first line of immune defense to a metabolic or physiologic insult, its dysfunction can compromise restoration of tissue homeostasis and function. Ethanol is known to cause pathological effects to the intestine, particularly the intestinal barrier. In our prior investigations, we found that tributyrin supplementation mitigates the negative effects of acute, chronic-binge, and chronic ethanol exposure on tight junction protein expression in the ileum and proximal colon and associated liver injury [27, 28]. Because macrophage number and butyrate yield are highest in the colon, we wanted to see whether these protective effects of tributyrin were linked with alterations in intestinal immune responses. Here, we present that the depleted innate immune response in the proximal colon following chronic-binge ethanol exposure is mitigated with tributyrin supplementation.

The mechanisms of these observations are likely multifactorial. Intestinal epithelial cells serve as the interface between the organism and environment and are therefore strategically positioned to signal environmental changes. In healthy intestinal mucosa, epithelial cells as well as mast and stromal cells produce and release TGF*β*, a potent monocyte chemokine, and IL-8, a neutrophil chemoattractant. Release of IL-8 is further induced after stimulation with IL-1*β* and lipopolysaccharide [35, 50]. Butyrate priming of intestinal epithelial cells has also been shown to enhance secretion of IL-8 [35]. Here, we demonstrate that ethanol alone has a negative effect *in vitro* on IL-8 secretion from intestinal epithelial cells (Caco-2), with or without stimulation with IL-1*β*, and that butyrate mitigated these effects, thus indicating a direct protective effect of butyrate. Additionally, ethanol-treated animals had reduced TGF*β* mRNA expression, but tributyrin treatment mitigated this effect. Stromal TGF*β* and IL-8 have been shown to recruit blood monocytes that express receptors for these chemokines [51]. Once recruited to the lamina propria, monocytes take up residence in the extracellular matrix to become resident macrophages. Constitutive expression of these chemokines by mucosal cells promotes ongoing recruitment of blood monocytes to the mucosa and, in combination with resident macrophages, makes the lamina propria in the gastrointestinal tract the body's largest reservoir for macrophages, with numbers highest in the colon [51]. We find here that in the presence of tributyrin, immune cell numbers are reflective of animals not exposed to ethanol and that ethanol greatly dampened immune cell presence in colonic lamina propria.

In pulmonary tissue, chronic ethanol exposure interferes with the actions of the granulocyte-macrophage colony-stimulating factor, which is secreted by various cells and stimulates the production of granulocytes and monocytes [52]. High levels of G-CSF are constitutively expressed in normal mouse or human intestine, and an exogenous commensal probiotic, *Lactobacillus rhamnosus*, was able to further enhance the expression [53]. In the absence of tributyrin, we found that ethanol-exposed mice had lower expression of G-CSF in the proximal colon. Coinciding with a reduction in complement factor, macrophages, and neutrophils, this could impact the ability for the host to clear cellular debris and potentially exacerbate ethanol-induced intestinal injury. Metalloproteinase-9 is linked with tissue regeneration, and G-CSF stimulation of neutrophils has been shown to increase the release of vascular endothelial growth factors and stimulate hind limb ischemic tissue regeneration [54]. Therefore, in our work, the reduction of MMP9 and depletion of alternatively activated macrophages in the mice only exposed to ethanol could contribute to downregulation of angiogenesis and potentially further inhibit infiltration of immune cells into the colonic villi. While high concentrations of butyrate are known to inhibit angiogenesis in tumor tissue, low concentrations of sodium butyrate have been shown to promote angiogenesis and tissue remodeling in tendon and bone injury [38].

Little is known about the effect of ethanol on intestinal vasculature, particularly in the colon. Ray et al. conducted a time study on the effects of intraluminal perfusion of 6% (wt/vol) ethanol on jejunal microvasculature and morphology in dogs [55]. They noted contraction of the villus core and compression of the lymphatics and concluded that these factors were the primary cause of ethanol-induced epithelial damage [55]. In the vasculature, the generation of superoxide anion and hydrogen peroxide induced by ethanol is associated with endothelial dysfunction, vasoconstriction, and hypertension [56, 57]. Reactive oxidant species scavenging attenuates the vascular dysfunction induced by ethanol [56]. Here, we present a depletion of endothelial markers vWF and CD31 in the proximal colon of mice only exposed to ethanol and that tributyrin supplementation enhanced endothelial marker expression during chronic-binge ethanol exposure. Vasodilation is known to be induced by short-chain fatty acids [58, 59]. Butyrate induced relaxation in small mesenteric arteries preconstricted with noradrenaline in rats, and this effect was found to be independent of intracellular pH and suspected to be linked with the cyclic AMP second messenger system [60].

## 5. Conclusion

In conclusion, these findings show that tributyrin supplementation protected against blunted immune responses, oxidative stress, and reduced vasculature in the mouse proximal colon caused by chronic-binge ethanol exposure. These data highlight beneficial effects of butyrate and suggest an important role of this gut fermentation byproduct as a potential protective supplement to ethanol exposure, and future studies investigating role in human models are warranted.

## Figures and Tables

**Table 1:** Primer sequences for real-time reverse transcription polymerase chain reaction.

Gene	Sequences (forward/reverse 5′-3′)	
	Forward	Reverse
18S	ACG GAA GGG CAC CAC CAG GA	CAC CAC CAC CCA CGG AAT CG
Arg1	CTC CAA GCC AAA GTC CTT AGA G	AGG AGC TGT CAT TAG GGA CAT C
CD45	GCA GTG CTA CGA GTG CTA TGG	ACT GAC GGG TCT TTA GTT TCC TT
CD68	CCA TCC TTC ACG ATG ACA CCT	GGC AGG GTT ATG AGT GAC AGT T
ELANE	CAG AGG CGT GGA GGT CAT TT	GAA GAT CCG CTG CAC AGA GA
GAPDH	AGG TCG GTG TGA ACG GAT TTG	TGT AGA CCA TGT AGT TGA GGT CA
HO-1	AAG CCG AGA ATG CTG AGT TCA	CGG GTG TAG ATA TGG TAC AAG GA
iNOS	GTT CTC AGC CCA ACA ATA CAA GA	GTG GAC GGG TCG ATG TCA C
MMP9	GCG CCA CCA CAG CCA ACT ATG	TGG ATG CCG TCT ATG TCG TCT TTA
NOX1	GGT TGG GGC TGA ACA TTT TTC	TCG ACA CAC AGG AAT CAG GAT
PDGFb	AAG TGT GAG ACA ATA GTG ACC CC	CAT GGG TGT GCT TAA ACT TTC G
SOD2	CAG ACC TGC CTT ACG ACT ATG G	CTC GGT GGC GTT GAG ATT GTT
TGF*β*	TGA CGT CAC TGG AGT TGT ACG G	GGT TCA TGT CAT GGA TGG TGC
TRX1	CAT GCC GAC CTT CCA GTT TTA	TTT CCT TGT TAG CAC CGG AGA

## Data Availability

Readers may access the data underlying the findings of this study by contacting the contributing author, Gail A. M. Cresci, at crescig@ccf.org.
